# How did the Treatment Work for Robin? And for Dylan? Studying Individual Youth Treatment Mediators Using Single-Case Experimental Designs

**DOI:** 10.1007/s10567-023-00442-7

**Published:** 2023-07-05

**Authors:** Marija Maric, Shawn I. Kok

**Affiliations:** 1 Research Institute of Child Development and Education, University of Amsterdam, Amsterdam, The Netherlands; 2 Department of Child Development and Education, Faculty of Social and Behavioral Sciences, University of Amsterdam, Postbus 15776, 1001 NG Amsterdam, The Netherlands; 3 University of Amsterdam, Nieuwe Achtergracht 127, 1018 WS Amsterdam, The Netherlands

**Keywords:** Treatment mediators, Single-case methods, Individual mechanisms, Youth interventions

## Abstract

Study of individual treatment mechanisms in youth interventions facilitates evidence-based development, selection and implementation of treatment components that are most effective for each individual child. This position paper aims to bring together two important topics from the area of youth intervention research: mediators of treatment outcomes and single-case experimental design methodology. We start by outlining the benefits of studying within-person mechanisms and propose how statistical mediation analysis and single-case methods can be integrated to enable this type of research. Further, we review existing methodology for the study of individual youth treatment mechanisms and provide recommendations for the clinical practice research.

In the 2012 volume of this journal, Maric et al. published a review on the importance of studying treatment mediation in youth intervention research. It was therewith emphasized that studying treatment mediators forms a crucial part in determining whether an intervention affects the intended symptom, risk factor or underlying process and hence whether it is effective (see also MacKinnon, 2008). Treatment mediators can thereby be seen as ‘mechanisms or processes through which a treatment achieves its effect’ (Kraemer et al., 2002). Furthermore, identifying treatment mediators can help to determine the most and least potent components of a treatment and thus further enhance its effectiveness and utility. Ultimately, this may decrease the mental burden that many children today are experiencing by allowing for treatments to become more adapted to their unique situation.

One section of the earlier review by Maric et al. (2012) was devoted to drawing attention to studying treatment mediators in single-case experimental designs (SCEDs). At that time there was, however, not much to be mentioned except that we found it important to study mediating variables not only on the level of a group (as in randomized controlled trails [RCTs]), but also on the level of an individual client. Now, over a decade later, the topic of assessing mediators in SCEDs has become more relevant than ever. The aims of this position paper are therefore to: (i) define the relevance of studying youth treatment mediators on the level of a single client; (ii) discuss how statistical mediation analysis and single-case methods can be integrated to facilitate this research; and (iii) review existing methodology for the study of individual youth treatment mechanisms and provide recommendations for the clinical practice research.

## Why is Attention for Individual Treatment Mediators Necessary?

There are at least three, somewhat interrelated, reasons to study treatment mediation in individual young clients. First, mental health problems in youth are not only highly prevalent (Merikangas et al., 2010; World Health Organization, 2022), but are also hard to treat due to complex risk factor interplay. Various (combinations of) child, parent, family, and social context mechanisms can cause and maintain mental health problems in children and adolescents. In child A., for example, an interaction between negative self-esteem, changing schools, and cannabis use leads to depression, while in child B. an inhibited temperament in combination with poor social skills and parental psychopathology leads to the same problem. This heterogeneity in risk factors and mechanisms is not only causing extreme suffering in young people, but also points to the need to utilize personalized youth interventions. Discovering individual treatment mediators facilitates the development, selection and implementation of treatment components that are most effective for each individual client.

Second, our field has made a tremendous progress in the past 60 years regarding developing and testing interventions for various youth and family conditions. An empirical base of these interventions was also extensively established so that we have now, for example, learned that different modalities of Cognitive Behavioural Treatment (CBT) are effective for youth anxiety, major depression and trauma (e.g., Ewing et al., 2015; Kreuze et al., 2018; Oud et al., 2019; Sigurvinsdóttir et al., 2020; Weems & Neill, 2020). At the same time, however, we have also learned that not all youth profits equally when following treatment (Robinson et al., 2020). Disentangling reasons for this has turned out to be a difficult task considering the designs used to study efficacy and effectiveness of these interventions.

RCTs have long been the gold standard to evaluate the effects of youth treatments. However, to determine the effects of an intervention in RCTs, average symptom scores of treatment groups are compared to average symptom scores of a control group. Next to this, meta-analyses of RCTs are often used to underpin a treatment evidence-base, averaging these effects over different groups even further. On the one hand, RCTs are therewith suitable to study intervention effects in large-scale (e.g., prevention or school-based programs), answering questions such as ‘is intervention A *on average* more effective than intervention B?’. On the other hand, however, averaging participants’ scores and effects sizes (as in meta-analyses) is problematic and leads to loss of valuable information about individual variations in treatment effects. As mentioned, youth mental health problems are heterogeneous in nature and treatment effects that evolve within-persons cannot be captured by between-persons comparisons (Maric et al., 2023; Schuurman, 2023).

The third and final reason we want to mention in the context of this paper is that studying mediators on an individual level can aid the research in real-world settings and populations. It can assist clinicians and other mental health professionals in learning how the treatment works for their individual clients and thus also other clients with similar profiles in similar circumstances. Furthermore, it provides a relatively easy way for researchers to test interventions and theories in concrete practical situations. All in all, this will certainly contribute to more evidence-based work of the therapists and more evidence-based clinical practice.

## How SCEDs may Help in Assessing Individual Treatment Effects?

In the past decades, SCED methods have regained in popularity as a way to test treatment effects in youth populations (Kazdin, 2019). SCEDs therewith provide within-individual comparisons in which symptoms of interest of one or several participants are tested regularly (e.g., monthly, weekly, daily, and/or hourly) over a period of time. Examples of SCED designs include (1) an AB design (baseline phase A followed by an intervention phase B), (2) A_1_B_1_A_2_B_2_ design (in which an intervention is withdrawn during A_2_ and again introduced during the B_2_ phase), and (3) the multiple baseline SCEDs in which clients are randomized to different lengths of a baseline phase A before introducing an intervention phase B, making it possible to account for maturity effects in clients or passage of time (Barlow et al., 2009; Tate et al., 2016).

**Fig. 1:**
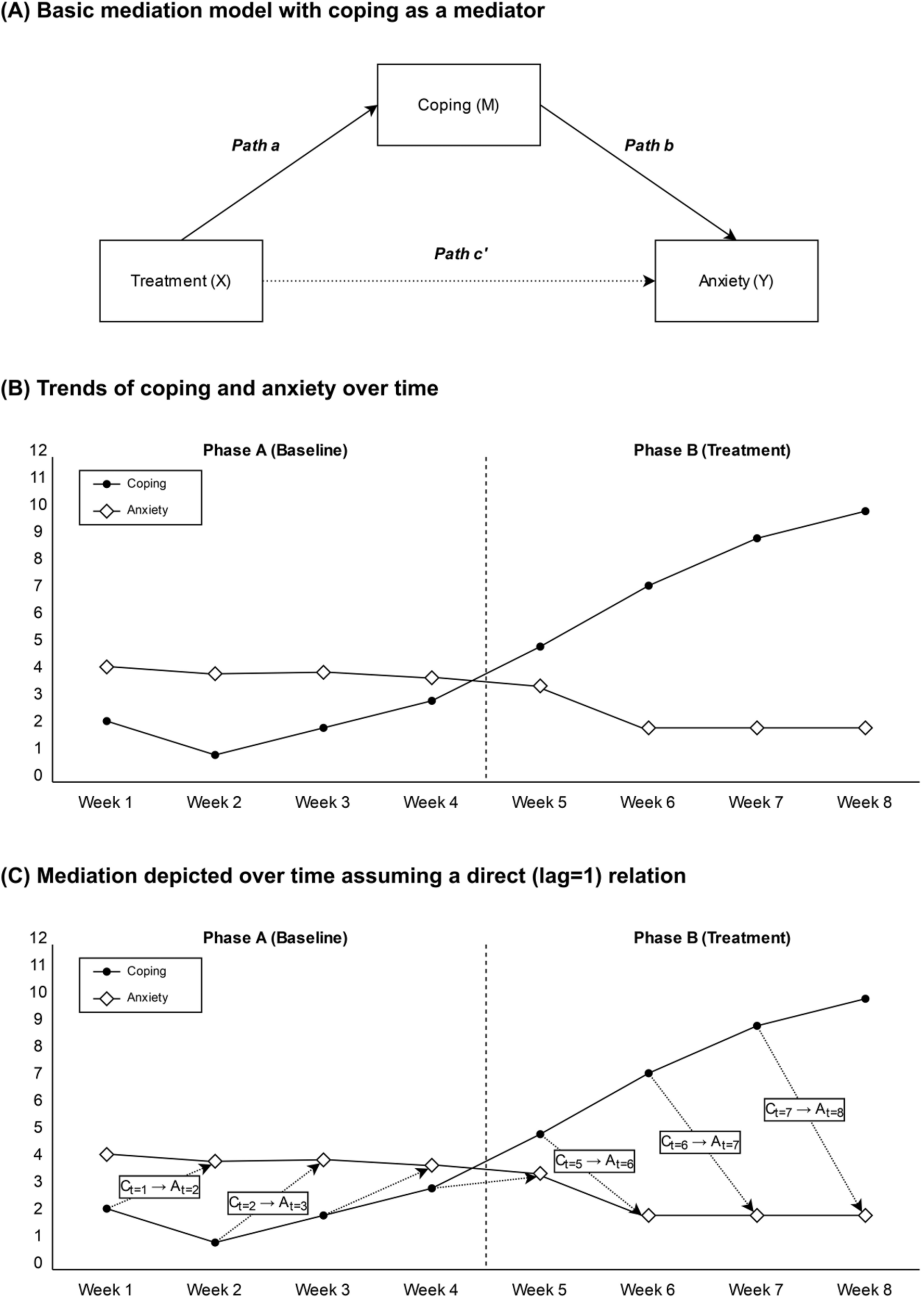
Basic display of SCED mediation analysis. Panel **a** depicts the single mediator model. Applying this methodology on the example depicted in panel (**b**), panel (**c**) shows a graphical representation of a mediation effect of coping between a treatment and anxiety assuming a direct (lag = 1) effect

An important advantage of SCEDs is that, through the introduction of different phases of the study, individual clients serve as their own control. Therefore, a matching control group is not necessary. In Fig. 1B, for example, anxiety symptoms and coping of a single participant were monitored during the baseline and treatment phase. This design helps to answer the question whether there were changes in anxiety after the introduction of the treatment compared to the baseline phase. Likewise, we can assess whether the individual’s coping changed after the introduction of the treatment. To a certain degree, this will allow us to make some preliminary predictions regarding the potential of coping as a treatment mediator. Nonetheless, as we will demonstrate below, a proper evaluation of such a relation requires a more sophisticated approach.

## Integrating Treatment Mediation in Youth Single-Case Research: Current Developments

The assessment of treatment mediators in SCEDs requires that two (or more) variables are tested simultaneously throughout each phase. Similar to group-level studies, the treatment mediator is therewith seen as a variable that lies within the causal chain between the intervention and (dependent) outcome (MacKinnon, 2008). An example of this relational model in SCEDs is depicted in Fig. 1A, in which a treatment (*X*) is hypothesized to produce changes in coping abilities (*M*), which in turn lead to changes in anxiety (*Y*). The effect of the treatment mediator can thereby be evaluated by statistically testing the effect of the treatment through paths *a* and *b*. It is important to note that the requirements of statistical mediation analysis which have previously been outlined for group studies (e.g., temporal precedence; Kazdin & Nock, 2003; MacKinnon, 2008; Maric et al., 2012) also apply when assessing mediators in SCEDs. Unlike group studies, however, SCEDs provide a within-subjects structure of data whereby each variable is repeatedly measured. This makes that the traditional mediation techniques may not be directly applied, and hence other data-analytic techniques are warranted.

While in the past decades attempts have been made to develop methodology to evaluate treatment mediators in SCEDs, each of them presented serious shortcomings. Gaynor and Harris (2008) designed a study to investigate whether changes in activation mediated changes in depressive symptoms in adolescents. The theoretical framework and the design were developed well, but the data were investigated visually. Although providing useful insights, visual inspections are nonetheless prone to bias and do not allow for a systematic evaluation of the extent to which the treatment mediator affects the outcome. In another study, Borckardt et al. (2008) tested changes in two processes over time, social engagement and mood, using cross-correlations. While correlations can be used to test associations in two variables over time, they are not a formal test of mediational pathways and treatment mediation. Finally, and more recent, Geuke et al. (2019) investigated the utility of combining previous SCED methodology to test for mediation and found that none of the techniques could be adapted to evaluate the *b* (the effect of the mediator on the outcome) and *c’* paths (the remaining effect of the treatment that is not accounted for by the mediator). Hence, further urging the need for new methodology to be developed.

Fueled by the Lorentz Workshop (2019), organized by Maric and colleagues in the city of Leiden in the Netherlands, preliminary efforts were made by a select group of clinical researchers and methodologists to develop methods to overcome these aforementioned shortcomings. Most of the proposed techniques consisted of piecewise linear regression analyses combined with interval testing for the obtained estimate of the mediated (or indirect) effect (Loeys & Rodenburg, 2022; Valente et al., 2022). In these techniques, each of the paths depicted in Fig. 1C are determined by assessing the change between phases in the averages (i.e., level), trends within the phase, or both. Furthermore, MacKinnon et al. (2022) described a randomized permutation test for single subject mediation using alternating treatment designs in which residuals are reassigned in many (i.e., thousands) possible combinations. This to acquire a sampling distribution which allows to test the likelihood of the mediated effect found based on the actual data. In a study published in another journal, piecewise regression analysis was again applied, and it was emphasized that, similar to large group treatment mediation studies, it is important to study variables next to the mediator and outcome that may confound this relation (Valente et al., 2022).

Simultaneous to this development of new techniques, attention was also provided to the handling of autocorrelation, or the tendency of repeated measures to be correlated with one another (Busk & Marascuilo, 1988). For example, if a child shows a decline in symptoms at the start of the treatment phase, it is more likely that these symptoms will keep decreasing in subsequent measures. In other words, a value at one time point is predictive for the subsequent measures. This may have serious implications, however, for the underlying statistical assumptions and may in turn lead to biased estimates of standard error and hence inaccurate findings (Kazdin, 2011). In mediation analysis specifically, the computation of the intervals may be significantly influenced as these are dependent on the acquired standard errors. Therefore, methods have been proposed to take autocorrelation into account when assessing treatment mediation in SCEDs.

One common practise is to add lagged values of the mediator and outcome as covariates when determining the path values (MacKinnon et al., 2022). These lagged values can either be directly preceding (i.e., the previous measure) or more delayed (e.g., the measure from two or more time points ago) depending on the expected autocorrelation. Another method has been proposed by Loeys & Rodenburg (2022) in which Generalised Least Squares (GLS) models are used to compute an autocorrelation structure similar to multilevel approaches. Like with the lagged covariates, different orders of autocorrelation can be accounted for by fitting a corresponding structure. Moreover, the GLS method allows for the assessment of a small set of individuals at the same time. Thus, providing a means to compare individual trends with the total image on a statistical level.

## Recommendations for the Youth Clinical Practice Research

Applying statistical mediation analysis in youth intervention research can be, in general, considered a challenging endeavor. Fortunately, established guidelines exist (Kazdin & Nock, 2003; MacKinnon, 2008; Maric et al., 2012) that can also be applied to design and conduct a study on treatment mediators in SCEDs. Because of the repeated structure of SCED data, existing statistical mediation data-analysis techniques do not apply and in this position paper we shared recent knowledge on the more suitable techniques. We conclude by proposing guidelines for a set of issues that emerged from youth clinical practice research. One challenge, for instance, comes with the number of observations that is required to be able to obtain valid results. It has previously been estimated that between 30 and 100 observations are required to detect large effects depending on the type of technique used (Loeys & Rodenburg, 2022; Valente et al., 2022). This, however, is difficult to achieve within the clinical research field, as most treatments do not lend for such an extensive treatment window or the burden of repeated assessments for the clients may be too extensive. Our advice is to use short, personalized, assessments of hypothesized mediators and outcomes in the study and to choose smaller intervals (e.g., daily or twice-weekly assessments). Another issue concerns temporal precedence requirement for mediation, i.e., changes in the mediator should precede and influence changes in the outcomes. Temporal precedence can be achieved through shifting outcome and mediator measurements one (or more) lags to the left (Fig. 1C) or through choosing to conduct measurements directly after each other (e.g., as in the Ollendick et al., 1995 study). Finally, it should be considered that different SCED designs may be more or less suitable for the study of different types of mediators, and that we should keep account of the potential interplay between different SCED designs, types of interventions, and mediators. For now, multiple baseline SCEDs seem as the most feasible and experimental single-case design that can be implemented in youth intervention research. This design lends itself also for studying the changes in mediators and outcomes in the intervention phase as opposed to the baseline phase.
